# A novel assessment, diagnostic and treatment system for diabetic foot

**DOI:** 10.1186/s13047-023-00687-z

**Published:** 2023-11-25

**Authors:** Jordi Viadé, Maria Nicolás, Magdalena Bundó, Marc Sirvent, Clàudia Riera, Miquel Sabriá

**Affiliations:** 1 Department of Endocrinology & Nutrition, Hospital Germans Trias I Pujol, Autonomous University of Barcelona, Badalona, Spain; 2 Private Practice Diabetic Foot Clinic, Autonomous University of Barcelona, Sabadell, Barcelona, Spain; 3 Primary Health Care Center Ronda Prim, Gerència d’Àmbit d’Atenció Primària Metropolitana Nord de Barcelona, Institut Català de La Salut, Mataró, Spain; 4 Department General, University Hospital of Granollers, CIBERCV, ISCIII, Granollers, Spain; 5 Department of Angiology and Vascular Surgery, University Hospital Germans Trias I Pujol, Badalona, Barcelona, Spain; 6 Department of Medicine, Autonomous University of Barcelona, Barcelona, Spain

**Keywords:** Diabetic foot, Ulcers, Neuropathy, Neuroischemia, Classification

## Abstract

**Background:**

This report aims to present a novel system for the management of foot lesions in patients with diabetes. It was developed in the diabetic foot unit (DFU) of the Mutua de Terrassa University Hospital (HUMT) by primary care professionals, the Diabetic Foot Clinic (DFC), and during emergency cases treated by our group based on daily activities in patients with neuropathy or neuroischemia.

**Body:**

This system considers five degrees of action based on two fixed variables: presence of infection and lesion depth. These two variables allowed the user to investigate aspects of the system until the overall action required by the pathology is made clear. These variables establish pathology stages of various severities that require different actions in aspects of care, management and treatment.

**Conclusion:**

This tool facilitates diagnosis, treatment, and coordination among different members of a multidisciplinary team working in specialized hospital units, primary care centers, and emergency settings.

## Background

Diabetic foot (DF) is a major cause of morbidity and mortality in patients with diabetes mellitus (DM) [1]. Most patients develop foot ulcers after age 40, and the risk increases with age [2]. The most comprehensive definition of DF, in our opinion, is “the presence of signs, symptoms, or foot ulcers as a result of chronic complications of diabetes” [3].

DF has a multicausal origin and is progressive. Deformities or changes in plantar pressure distribution, a general lack of sensitivity and the presence of an underlying vegetative and vascular disorder, can all accelerate fissuring of the horny layer. This can create a lesion that progresses toward deeper soft tissues and can reach the bone [3–5], putting the limb and even the life of the patient at risk [6].

Multiple classifications or scales have been published to establish the risk and severity of DF-related injury, or as a means of communication between professionals [7–9]; however, there is currently no universally-accepted classification [9].

We present a system for the evaluation and treatment of foot lesions in patients with DF that facilitates diagnosis and treatment, as well as coordination and management between members of multidisciplinary teams and healthcare professionals in primary and/or emergency care centers. It has been used by researchers for over 20 years, and its implementation in a multidisciplinary team has led to a considerable reduction in the number of major amputations [10].

## The system

In 1992, a diabetic foot unit (DFU) was established at Mutua de Terrassa University Hospital (HUMT), comprising an endocrinologist, a nurse educator, and a specialist podiatrist. To facilitate coordination between the different care areas, a system was created to evaluate and treat foot complications in patients with DF. Over years of continuous training, clinical practice, and with the contributions of certain specialisties in the vascular surgery and infectious diseases unit, the system was further expanded. Beginning in 2013, the German Trias i Pujol Hospital organized a multidisciplinary DF team and decided to implement the evaluation and treatment system used in HUMT for its operations as well. More specialists were incorporated including plastic surgery, diagnostic imaging, traumatology, home care, nutrition, social work, and rehabilitation. With the collaboration of these diverse specialties, a revision and expansion of the system was carried out (Table 1).

**Table 1:** Diabetic foot evaluation and treatment system

Ulcer grade	0	1	2	3	4
**CHARACTERISTICS**					
**DEPTH**	No ulcer	Epidermis/Dermis	G1 + Subcutaneous tissue	G2 + Fascia/Muscle/Bone	G3 + Critical ischaemia? Yes/No
	Warmth, oedema, erythema. DISMISS	Signs of ischemia^a^	Signs of ischemia^a^	Signs of ischemia^a^	and/or Necrosis areas
**INFECTION?**	Assess	No	Superficial	Deep/Located	and/or Systemic toxicity
**EVALUATION**	Neuroischemic screenig Skin thermometry	Neuroischemic screening	Neuroischemic screening	Neuroischemic screening	Neuroischemic screening
	Charcot Neuro-arthropathy? Image diagnosis		Microbiological Culture	Microbiological Culture	Microbiological Culture
			Probe to bone	Probe to bone	Probe to bone
**TREATMENT**	Suspicion of Charcot neuro-arthropathy:			Debridement, surgery	Debridement and/or Revascularization
			Oral antibiotic	Oral antibiotic/IV	Antibiotic IV
	Tie up	Dressings	Dressings	Dressings	Dressings
	Surgery?	Offloading	Offloading	Offloading	
				Relative rest	Absolute rest
**CARE LEVEL**	Primary care/ DFU/ Hospital	Primary care	Primary care/ DFU	DFU/ Hospital	Hospital/ DFU
**DIABETES EDUCATION**	Review	Review	Review	Review	Review

*G* Ulcer Grade, *IV* intravenous, *DFU* Diabetic Foot Unit

^a^Signs of ischemia = Consider one more grade

The system considers five degrees of action based on two fixed variables: the presence of infection and the lesion depth. Both allow users to investigate other aspects of the system until the overall action required by the pathology is made clear. These variables establish DF stages of different severities that require different actions in terms of care, management, and treatment.

The evaluation includes important aspects such as neuroischemic screening, microbiological culture, probe to bone or image scans to rule out the presence of osteomyelitis or neuroarthropathy. It also includes the different therapeutic actions recommended for each stage or grade, ranging from the type of offloading to topical wound dressings or oral antibiotics.

The complete system table (Table 1) shows the most appropriate level of treatment depending on the degree of DF, as well as a reminder regarding the importance of diabetes education. The podiatrist or nurse educator provides support care for hygiene measures, hydration, recognition of warning signs, and footwear—all of which help avoid recurrences.

Grade 0 rules out the presence of Charcot neuroarthropathy or underlying infections without ulcerations. This grade is used for the diagnosis of phase 0 of Charcot foot or infection without ulceration. If there is an ulcer, the following grades are used: grade 1 for non-infected superficial ulcers; grade 2 for ulcers that reach the subcutaneous tissue with signs of superficial infection; grade 3 for ulcers that reach the fascia, muscle, and/or bone with localized deep infection; and grade 4 for those with systemic involvement, critical ischemia or necrosis.

Patients with insufficient blood supplies should be considered and treated as higher-grade, because the presence of arterial ischemia worsens the prognosis [9].

This system table includes eight complementary tables (neuroischemic screening, diagnosis of Charcot neuroarthropathy, sample collection for microbiological culture, diagnosis of osteomyelitis, offloading systems, oral antibiotics, topical treatment, and surgical techniques) that add to content and help determine generalized treatments.

## Discussion

The goal of developing this system was to guide healthcare professionals, from primary care to the emergency room, in the generalized management of patients with DF who have suspected Charcot neuroarthropathy or foot ulcers, enabling them to act quickly [11] and thus maximize their chances of avoiding complications or the need for amputation.

Our system was not intended to be compared to other published classifications; it simply aims to offer a new tool for the management of this health problem, and has proven very useful in our DFU for a long period of time. As part of our tool comprises wound classification, we wanted to discuss the other most commonly used classifications currently used. In the scales published to date, certain essential aspects of clinical management have not been considered. For example, the wound depth, ischemia, and foot infection (WIFI) system [12] is a good option for assessing the level of ischemia and the benefit of revascularization; however, it does not include neuropathy, ulcer location, or ulcer extension. The International Working Group on Diabetic Foot (IWGDF) [13] classification only considers infection and does not assess other parameters that are important for the prognosis of ulcers—such as ischemia, neuropathy, location, depth, and extension of the ulcer. Although the University of Texas classification [14] is commonly used, it does not consider neuropathy or ulcer areas, which are major determinant of healing. Perfusion, extent, depth, infection, and sensation (PEDIS) [15] may be the most complete, although it is not easy to use in daily clinical practice and is most appropriate for determining the inclusion and exclusion criteria for research projects [8, 16]. The IWGDF currently recommends using the site, ischaemia, neuropathy, bacterial infection, area, and depth (SINBAD) classification because it includes the largest number of ulcer characteristics, and because it is a simple scoring system that is easy to use by any specialist. However, it does not provide 100% specific information on lesion characteristics, which is why it has been criticized for being insufficient [9].

The involvement of a multidisciplinary team can improve the prognosis of DF [17, 18]. Coordination between departments that care for patients with DF is therefore essential for its proper management.

Because these patients must be treated by a multidisciplinary team, unifying all variants in a single document, and considering the most appropriate level of care at each time point, makes handling their cases easier.

In many countries, the first contact between a patient and the health care system is through primary care [1]—which is often responsible for prevention, early detection, or referral to secondary level.

Our novel system is a tool that facilitates coordinated work among multidisciplinary teams to achieve the comprehensive management of patients with DF and decrease major amputation rates. In HUMT, between 2003 and 2012 when the system was first adopted, major amputations decreased by 67%—from 34 major amputations in 2003 to 11 in 2012. In HUGTP, from 2013–2021, the number of major amputations decreased from 153 (2010–2014) to 71(2015–2020) [10, 19].

## Data Availability

Not applicable.

## Abbreviations

DF: Diabetic Foot
DFC: Diabetic Foot Clinic
DM: Diabetes Mellitus
DFU: Diabetic Foot Unit
HUMT: Mutua de Terrassa University Hospital
HUGTP: Germans Trias I Pujol University Hospital
G: Ulcer grade
G1: Ulcer grade one
G2: Ulcer grade two
G3: Ulcer grade three
LOPS: Loss of protective sensation
WIFI: Wound depth, ischemia and foot infection
IWGDF: International Working Group on Diabetic Foot
PEDIS: Perfusion, extent, depth, infection, and sensation
SINBAD: Site, ischaemia, neuropathy, bacterial infection, area, and depth
